# Compound changes in temperature and snow depth lead to asymmetric and nonlinear responses in landscape freeze–thaw

**DOI:** 10.1038/s41598-022-06320-6

**Published:** 2022-02-09

**Authors:** Shadi Hatami, Ali Nazemi

**Affiliations:** Department of Building, Civil, and Environmental Engineering, Concordia University, 1455 De Maisonneuve Blvd. W. Montréal, Quebec, H3G 1M8 Canada

**Keywords:** Climate-change impacts, Hydrology, Projection and prediction

## Abstract

Cycles of freeze–thaw (FT) are among the key landscape processes in cold regions. Under current global warming, understanding the alterations in FT characteristics is of a great importance for advising land management strategies in northern latitudes. Using a generic statistical approach, we address the impacts of compound changes in air temperature and snow depth on FT responses across Québec, a Canadian province ~ 2.5 times larger than France. Our findings show significant and complex responses of landscape FT to compound changes in temperature and snow depth. We note a vivid spatial divide between northern and southern regions and point to the asymmetric and nonlinear nature of the FT response. In general, the response of FT characteristics is amplified under compound warming compared to cooling conditions. In addition, FT responses include nonlinearity, meaning that compounding changes in temperature and snow depth have more severe impacts compared to the cumulative response of each individually. These asymmetric and nonlinear responses have important implications for the future environment and socio-economic management in a thawing Québec and highlight the complexity of landscape responses to climatic changes in cold regions.

## Introduction

Freeze–thaw (FT) dynamics, i.e. the fluctuations of soil state between frozen and thawed conditions^1^, is among the most important land-surface characteristics in northern regions, playing a major role in determining soil properties^2^, hydrologic response^3^ as well as ecosystem diversity and productivity^4^. Due to these critical impacts, FT dynamics are key considerations for human activities such as agriculture^5^ as well as the construction and operation of infrastructures in cold regions^6^. Heightened climate variability and change, however, have significantly affected soil temperature patterns and consequently the dynamics of FT cycles^7^. Changing climate can impact FT patterns in several ways. Increasing air temperature, for instance, can affect the FT dynamics through decreasing the length of frozen season^8^, increasing depth of the active soil layer^9^, and permafrost retreat^10^. Decreasing snow depth, in parallel, reduces the thermal insulation of soil interface with atmosphere and increases soil vulnerability to fluctuations in air temperature^11,12^. This can in turn contribute to decreasing frost depth and increasing the frequency of swings in FT states^13^. Such changes can result in widespread alterations in regional hydrology^14^, phenology^15^, geology^16^, water quantity, and quality^17^ as well as socio-economic activities^18^. In addition, some of these alterations can create feedback effects with other elements of the environment. For instance, thawing landscapes in northern regions can affect the climate system through the emission of excessive greenhouse gas fluxes^19^, which can intensify the rate of global warming^20^. These impacts together pose various challenges to northern communities, where not only natural processes and socio-economic activities but also cultural values and unique ways of life are strongly tied with the dynamics of FT cycles^21^.

One example of such a region is the province of Québec in Canada. Spanning from 57° 15′ to 79° 23′ west and 44° 59′ to 62° 09′ north, Québec is the largest Canadian province with a total area of 1,542,056 km^2^. 176,928 km^2^ of this area is covered with freshwater bodies, marking Québec the richest Canadian jurisdiction in terms of surface freshwater availability. By including eight out of 15 Canadian ecozones, Québec is also one of the richest Canadian regions in terms of ecosystem diversity^22^. Almost the entire area of the Québec’s landmass undergoes three states of FT during a typical year, including continuous periods of frozen and thawed states, divided by a transient period, in which the landscape lingers between frozen and thawed conditions throughout a diurnal cycle. Having said that, as the area is massive and landscape characteristics are diverse, regional FT characteristics are subject to large spatial variability^23,24^. This becomes crucial in light of significant alterations in regional temperature and snow depth^25^, the two most influential climatic controls of FT at larger temporal and spatial scales^8,11,16^. At this stage, similar to many other cold regions, it is not yet clear how FT cycles across Québec respond to individual and compound changes in temperate and snow depth. This is a major gap as immediate management decisions are required to face the consequences of the thawing landscape in Québec and other regions in Canada, Alaska, Russia, and Northern Europe.

The knowledge gap in accounting for the FT response to changing climate stems from different sources. Firstly, most of our current understandings about the landscape response to climate change are based on in-situ data that are rather sparse spatially and discontinuous temporally^8,26^. Secondly, in order to quantify the impact of changing climate on FT dynamics, physically-based approaches, implemented in the current generation of land-surface schemes, are typically used. These models mostly involve the coupling water and energy balance, above, at, and below the surface^27,28^. Despite current advancements, available assessment frameworks are rather incomplete due to limitations in both data availability and modeling capability^29^. While in-situ data provide valuable information on the local control of climate on FT dynamics, the lack of data, especially in higher latitudes, poses significant constraints on the ability of in-situ data to capture the characteristics of changing FT patterns in time and space^30^. In addition, despite the fact that physically-based models are theoretically sound, they often suffer from oversimplified process representations and require large data supports that are often unavailable.

We argue that recent advancements in remote sensing technology along with the advent of powerful statistical tools can address some of the above limitations. On the one hand, satellite remote sensing data can overcome the limitations in in-situ observation of FT state at larger spatial and temporal scales by providing a synoptic and continuous monitoring of FT dynamics. This can provide an opportunity to systematically inspect temporal and spatial dependencies in FT dynamics, and their dependence with relevant climate variables^16,31^. On the other hand, if the purpose of modeling is shifted from continuous representations of FT states to representations of FT characteristics in time and space, then various statistical frameworks with much more flexibility can be used to describe functional links between FT and climate characteristics.

One statistical approach of such kind is the copula methodology, a formal framework to represent statistical dependence, which is widely used in recent hydrologic and environmental studies^32–34^. Copulas can provide a generic solution to describe the impacts of individual and compound changes in temperature, snow depth, and other relevant variables if needed, on FT characteristics through conditional modeling. In the context of FT modeling, copulas have been recently used for characterizing the link between FT states and near surface air temperature^35^. Here we also account for the effect of snow cover. The data support for setting up such frameworks is currently available through various publicly available gridded data^36–38^. Vine copula methodology was also tested recently for accounting the compound impacts of temperature and snow depth on FT characteristics^24^, which allows us here to set up a bottom-up impact assessment framework, with which the impacts of changing climatic conditions on FT characteristics can be quantified systematically across different spatial and temporal scales—see more details in “Methods” below.

## Results

**Figure 1:**
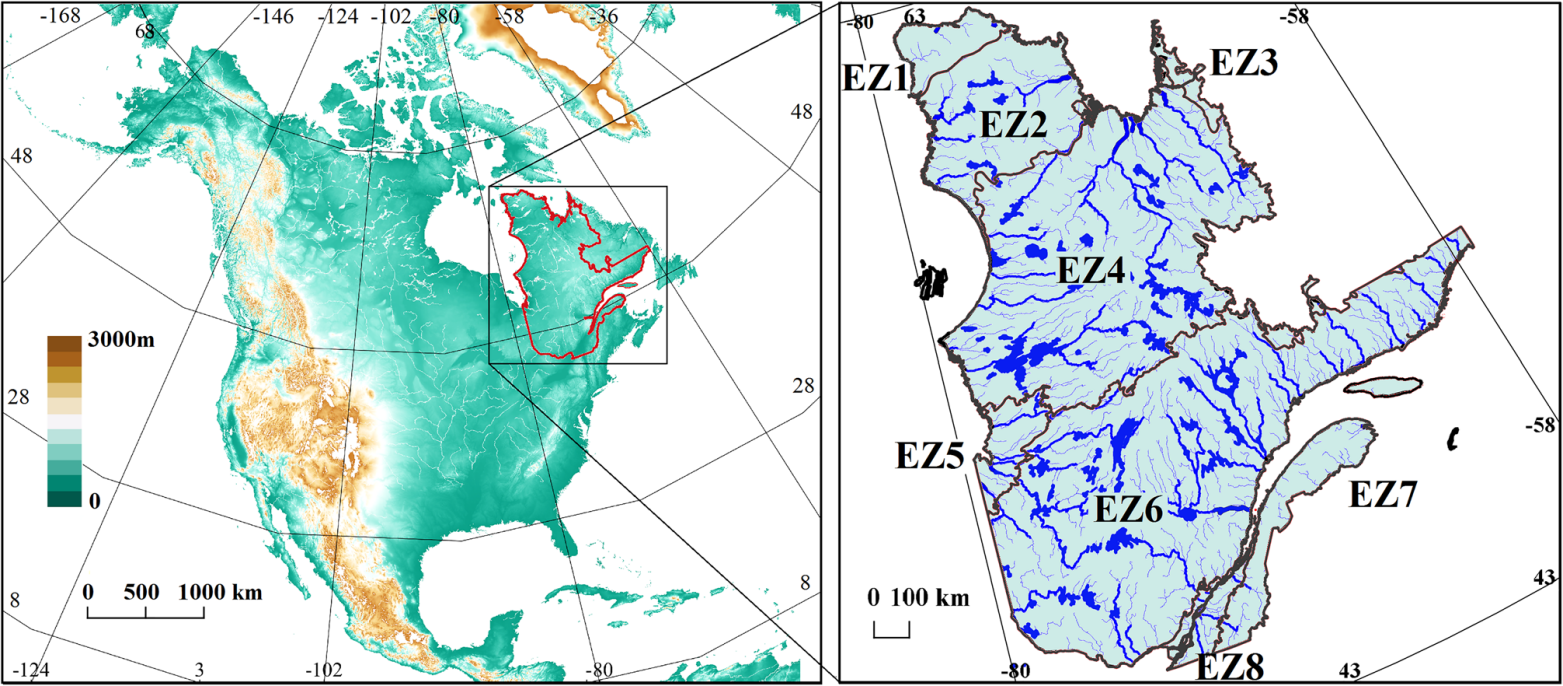
The province of Québec in Canada and its eight ecozones. This map is created by the authors using the QGIS v2.18.15 open-source software (qgis.org).

Québec includes eight ecozones that are regions with similar land, soil, vegetation, and climatic characteristics^39^. From the north to south, these ecozones include Northern Arctic (EZ1), Southern Arctic (EZ2), Arctic Cordillera (EZ3), Taiga Shield (EZ4), Hudson Plains (EZ5), Boreal Shield (EZ6), Atlantic Maritime (EZ7), and Mixed Wood Plains (EZ8), respectively^40^—see Fig. 1.

In each ecozone, the annual number of Frozen Days (*FD*_*year*_) and the number of transient days in the winter season (December, January, and February; *FTD*_*DJF*_) at grid scale are extracted. *FD*_*year*_ is defined as the total number of days with frozen soil in both AM and PM of a given day, spanned from September 1st to August 31st. *FTD*_*DJF*_ is also defined as the total number of days with the transient state over a typical winter season (months December, January and February). Transient days are defined above as days with frozen soil in AM and thawed soil in PM, or vice versa (thawed in AM, frozen in PM). In line with earlier studies^41,42^, our empirical results also show that these characteristics are the two most sensitive regional FT characteristics to changes in climate conditions, and demonstrate the strongest interdependence with temperature and snow depth at common scales and across the eight ecozones of Québec. These FT characteristics have also direct implications for land management. The changing annual number of frozen days can affect the length of phenological and agricultural activities^43^, accessibility to natural resources^44^, and the emission of greenhouse gasses from thawing permafrost^45^. In addition, the increasing number of transient days can be a proxy for land subsidence and erosion^46^ as well as the deterioration rate of civil infrastructures such as buildings, roads, and pipelines^47^.

**Figure 2:**
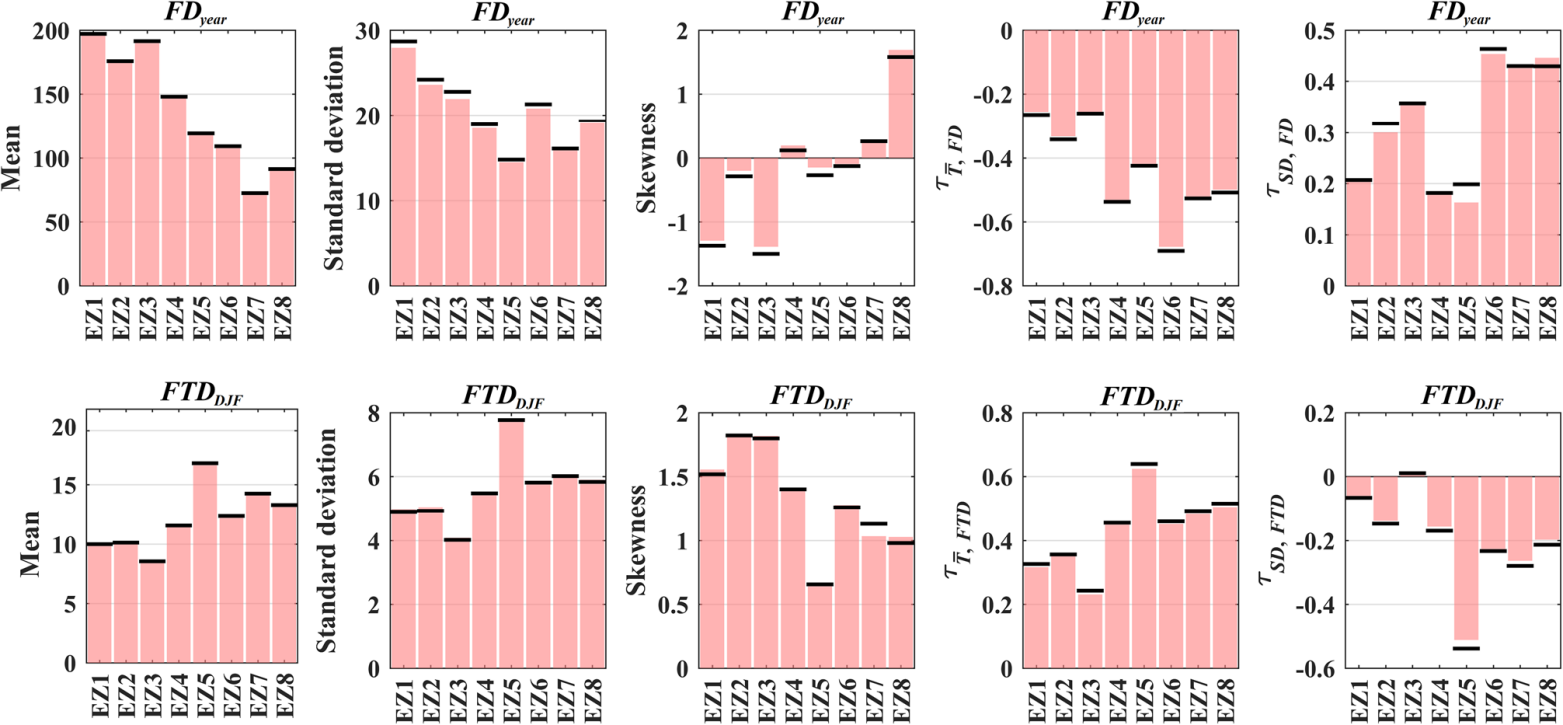
Observed (thick threshold lines) *vs.* simulated values (pink bars) for mean, standard deviation, skewness, as well as Kendall’s tau dependencies of FT characteristics with temperature and snow depth across eight ecozones of Québec during 1998 to 2016, displayed from left to right. The top and bottom rows depict the results for the number of frozen days in a typical year (*FD*_*year*_) and the number of transient days in a typical winter season (*FTD*_*DJF*_), respectively.

By applying the proposed framework (see “Methods” below), FT characteristics at each ecozone can be conditioned to air temperature and snow depth using developed copula models^48,49^. Before applying these models for impact assessments, we evaluate their performance in representing the marginal and joint characteristics of the two considered FT characteristics, observed across the eight ecozones. Figure 2 summarizes the results, in which the top row depicts the results for the annual number of frozen days (*FD*_*year*_) and the bottom row is related to the number of transient days during the winter season (*FTD*_*DJF*_). In each row, panels from left to right show the expected estimations (bars) and observed values (thresholds) for the mean, standard deviation, and skewness of the considered FT characteristics as well as their dependence with temperature and snow depth across the eight ecozones. Figure 2 clearly shows that the parametrized copulas can effectively represent the first three moments of the empirical probability distributions of both FT characteristics considered. For *FD*_*year*_, the overall expected relative error of 0.2%, 2.2%, and 13.5% is observed for mean, standard deviation, and skewness, respectively. For *FTD*_*DJF*_, the overall expected relative error for the first three moments are 0.4%, 1.1%, and 3.1%. The interdependencies between the considered FT characteristics with temperature and snow depth are also preserved with average relative errors of ~ 1% or less in all cases. Such performances in reconstructing the historical FT characteristics justify the application of the model for assessing the compounding impacts of changing temperature and snow depth.

**Figure 3:**
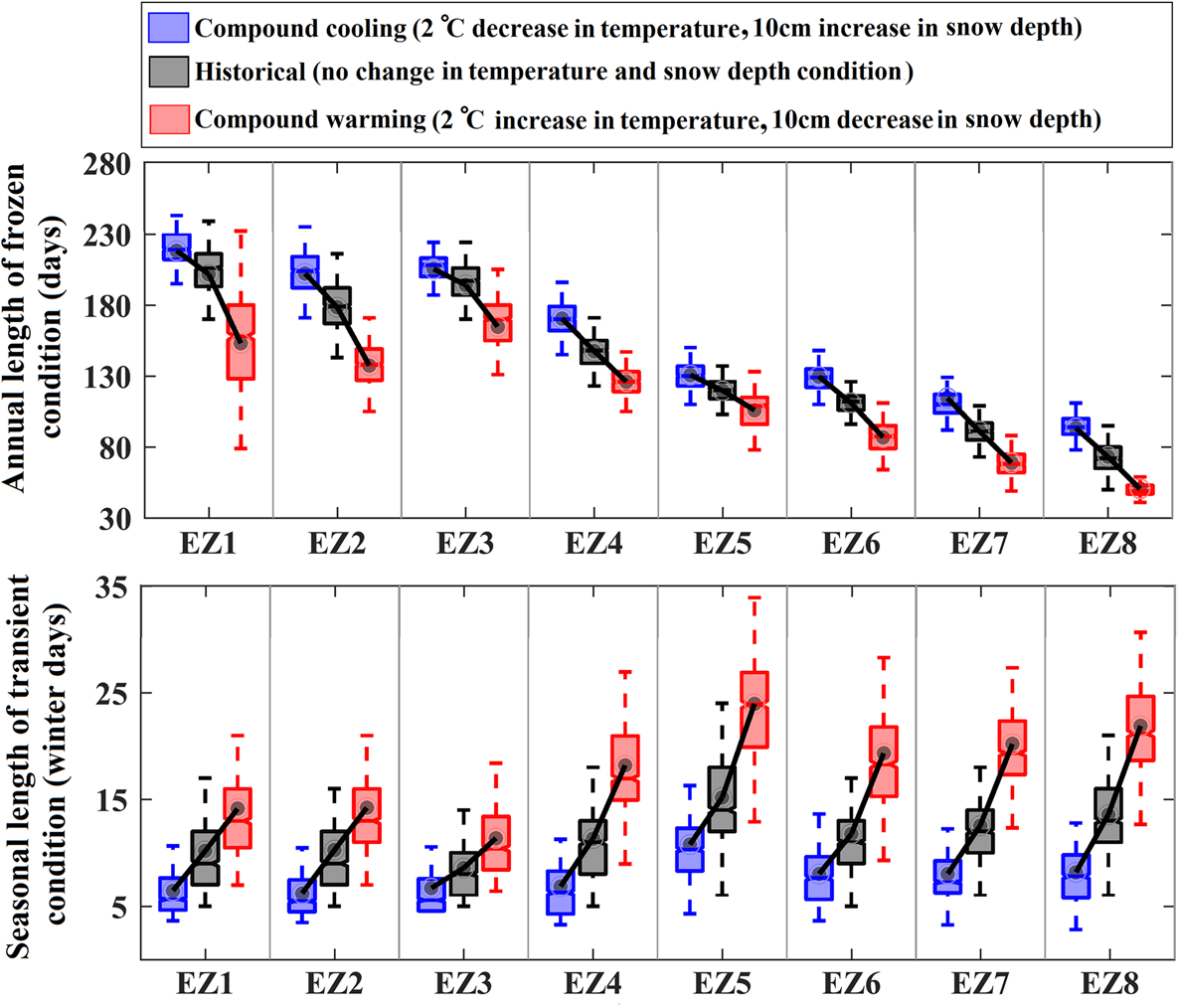
The response of *FD*_*year*_ (top row) and *FTD*_*DJF*_ (bottom row) to opposite compound scenarios of change in temperature and snow depth conditions. Historical as well as compound cooling and warming scenarios are shown with grey, blue and red boxplots obtained by 1000 resampling trials. Dots show the simulated mean ensemble of FT characteristics at each ecozone.

Apart from benchmarking the performance of copula models in reconstructing the historical dependencies between FT characteristics and climatic conditions, Fig. 2 shows a clear increase in the magnitude of dependencies between *FD*_*year*_ and both temperature and snow depth by moving from north to south. Similarly, the magnitude of dependence between *FTD*_*DJF*_ and the considered climatic drivers generally increases in southern ecozones. Using the one-way ANOVA test, we assess the differences between the estimated dependencies across different ecozones. The results highlight the uniqueness of interdependencies between *FD*_*year*_, temperature, and snow depth across all ecozones. In addition, dependencies between *FTD*_*DJF*_ and climate drivers are almost unique. The only statistically significant similarity is in the case of *FTD*_*DJF*_ and temperature in EZ4 and EZ6. Significant variations in the dependencies between FT characteristics and hydroclimatic drivers can reveal the effect of ecosystem conditions on regulating the impacts of changing conditions on FT characteristics. To showcase this empirically, we consider two compound scenarios related to opposing warming and cooling conditions, consisting of simultaneous changes of 2 °C warming in long-term mean temperature along with 10 cm thinner snow on the ground (compound warming), as well as 2 °C cooling in long-term mean temperature along with 10 cm thicker snow on the ground (compound cooling). Figure 3 summarizes the results of this impact assessment and compares them with historical conditions. Whiskers span the range of expected FT characteristics obtained by 1,000 resampling trials—see “Methods” for the details of the proposed impact assessment framework. Historical baseline (no change condition) as well as compound cooling and warming scenarios are shown with gray, blue, and red colors, respectively.

Using Fig. 3, a couple of key findings are made. First, there is a clear geographic departure between FT responses to unique compound changes in temperature and snow depth. While the response of *FD*_*year*_ to compound cooling and warming is more vivid in the north, the impacts on *FTD*_*DJF*_ are more pronounced in the south. Considering *FD*_*year*_ and under compound warming conditions, on average between 48 (EZ1) to 22 (EZ8) fewer days with the frozen condition are expected in a typical year. The north–south decline in the response of *FD*_*year*_ is less obvious under compound cooling conditions, ranging from roughly 10 (EZ5) to 34 (EZ2) days extension in frozen conditions per year. Considering *FTD*_*DJF*_, in contrast, the impact of compound warming increases by moving from north to south. Under this scenario and on average, 10 more days during a typical winter with the transient condition is expected in EZ8, where the majority of Québec’s population is concentrated, as opposed to only 5 days in EZ1. Under cooling conditions, this change ranges from roughly 2 (EZ3) to 6 (EZ8) fewer days with transient conditions in a typical winter. These spatially heterogeneous impacts on FT characteristics under unique compound changes in temperature and snow depth can reveal how changes in ecozonal features (e.g. vegetation and soil types, land cover, climate, etc.) regulate FT responses to compound changes in climate conditions.

Second, Fig. 3 clearly shows that in those ecozones in which FT characteristics are more sensitive to compound changes, responses to opposite warming and cooling scenarios become asymmetric and are more vivid under the compound warming scenario. For instance, looking at *FD*_*year*_, the absolute shift under compound warming scenario is 30 days more in the Northern Arctic (EZ1), compared to the shift under cooling scenario. The higher impact of warming on *FD*_*year*_ in northern ecozones is of great importance in the context of the land-atmospheric Carbon emissions due to permafrost degradation^50^. Similarly, in the case of *FTD*_*DJF*_, the magnitude of positive shift due to the compound warming scenario is about 4 days more in the Mixed Wood Plains (EZ8) compared to the negative shift caused by the compound cooling scenario. More sensitivity to the compound warmings has important implications regarding soil stability^51^ and deterioration of critical infrastructure^52^ that are rapidly aging^53^.

**Figure 4:**
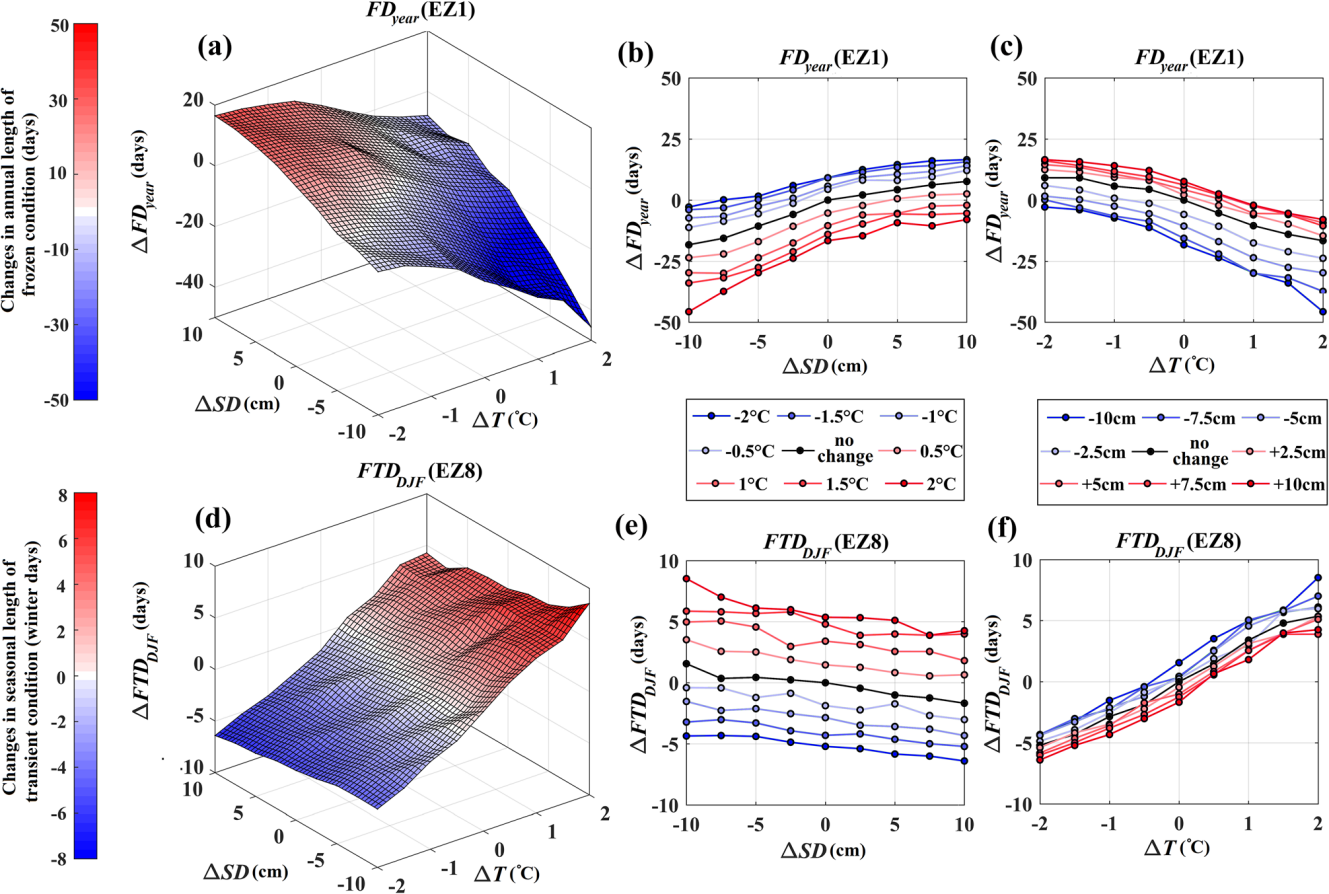
The response surfaces of *FD*_*year*_ in the Northern Arctic (EZ1, panel **a**) and *FTD*_*DJF*_ in the Mixed Wood Plains (EZ8, panel **d**) along with marginal sensitivity analyses to $\pm$ 2 °C change in temperature (panels **b** and **c**) and $\pm$ 10 cm change in snow depth (panels **e** and **f**) conditions.

To better understand climate controls on FT characteristics, we consider additional compound and individual changes in temperature and snow depth by mixing-and-matching long-term shifts in mean temperature, ranging from −2 °C to +2 °C (sampled every 0.5 °C), and mean snow depth, ranging from − 10 cm to 10 cm (sampled every 2.5 cm). This configuration results in 81 different scenarios, with which *FD*_*year*_ in the Northern Arctic (EZ1) and *FTD*_*DJF*_ in the Mixed Wood Plains (EZ8) are conditioned. We choose these two ecozones due to their largest sensitivity in changing hydroclimatic conditions with respect to *FD*_*year*_ and *FTD*_*DJF*_, respectively (see Fig. 3); and accordingly, they can better reveal how marginal and joint variations in temperature and snow depth can result in alterations of FT characteristics. Figure 4 presents the results of this analysis, in which the top (panels a to c) and the bottom rows (panels d to f) depict the results related to *FD*_*year*_ and *FTD*_*DJF*_, respectively. Panels a and d show response surfaces for changes in FT characteristics with respect to changes in temperature and snow depth. These response surfaces are reconstructed using the proposed C-vine copula and 1000 resampling—see “Methods”below. Panels b and e, as well as c and f, show the partial derivatives of these response surfaces, derived at known temperature and snow depth conditions, respectively.

Looking at the results obtained for *FD*_*year*_ across EZ1 (Fig. 4, first row), several interesting observations can be made. First and foremost, the non-symmetric response of FT characteristics to warming *vs.* cooling again reveals itself and is even more vivid in this round of analysis. While cooler temperature and increased snow depth increase the length of the frozen period in a typical year by around 18 days, warmer temperature and decreased snow depth can decrease the length of the annual frozen period by around 48 days. Projections of response surfaces, shown in panels (b) and (c), clearly present higher sensitivity of *FD*_*year*_ to warmer temperatures and thinner snow depth. Second, despite having different signs, the magnitude of interdependencies with temperature and snow depth is more or less the same (− 0.27 and 0.22, respectively; see Fig. 2); and therefore, the sensitivity of *FD*_*year*_ to changes in temperature and snow depth are rather similar*.* This is empirically the case. The expected change in *FD*_*year*_ under constant temperature conditions when snow depth is varying from − 10 cm to 10 cm of its long-term average value is about 25 days (Fig. 4b). Similarly, the expected change in *FD*_*year*_ under constant snow depth when the temperature is shifted from − 2 °C to + 2 °C of the annual long-term average is about 30 days (Fig. 4c). Having said that, it should be mentioned that the impacts of both snow depth and temperature variations are intensified as the temperature warms and snow depth declines. This is another line of evidence for asymmetric marginal impacts that are further controlled by the state of other driving variables.

A similar rationale can be used to interpret the results obtained for *FTD*_*DJF*_ across the Mixed Wood Plains (EZ8; the bottom row of Fig. 4). The changes in the expected *FTD*_*DJF*_ range from 9 more transient days under warmer climate with thinner snow depth to 7 fewer days with transient days under cooler climate and deeper snow depth. This observation again points at the asymmetric response of the FT characteristics, although it is less obvious compared to *FD*_*year*_ across EZ1. In this case, however, the dependence between *FTD*_*DJF*_ and temperature is stronger than the dependence between *FTD*_*DJF*_ and snow depth; and therefore, this FT characteristic is more sensitive to temperature changes, compared to changes in the snow depth. As an example, the expected magnitude of change in *FTD*_*DJF*_ is about 5 days under constant temperature conditions (Fig. 4e), while it is about 12 days under constant snow depth conditions (Fig. 4f). Similar to the case of *FD*_*year*_ across EZ1, the marginal impacts of change in snow depth are more intense under a warmer climate, ranging from 3 days under 2 °C cooling to 6 days under 2 °C warming. Although less pronounced, this is the case also for the marginal impacts of change in temperature under decreased snow depth conditions.

## Discussion

The results provided above illustrate two important findings. First, the impacts of changing temperature and snow depth conditions on FT characteristics differ spatially, with distinct manifestations and natures of response in northern and southern regions. Second, asymmetric responses to compound warming and cooling conditions are seen across different FT characteristics and/or regions. In general, when there is a significant sensitivity to changing climate, the response is more intense under warmer and/or decreased snow depth conditions. In this section, we look at another key feature of FT responses to compound changes in temperature and snow depth, which is rather overlooked in current literature. One key feature of the response to compound events is the nonlinear nature of the response, meaning that the impact of compound events can be more intense compared to the cumulative impacts of individual events when occurring independently^54,55^. Here we formally address the nonlinearity in FT responses at the grid resolution, by inspecting the deviation from the superposition principle of linear systems. This is through simulating FT responses to historical baseline and six opposing individual and compound scenarios of change in temperature and snow depth. Table 1 summarizes these scenarios. We address the non-linearity in FT responses by comparing the expected values of FT characteristic under individual and compound changes in temperature and snow depth, and under cooling and warming conditions.

**Table 1:** Individual and compound climate scenarios considered for addressing the nonlinear response of FT characteristics to individual and compound changes in temperature and snow depth conditions across Québec and at the grid scale.

Scenario description	Type	Notation
Long-term means of temperature and snow depth	Historical baseline (no-change)	(0, 0)
2 °C cooler compared to the long-term mean	Individual (cooling only)	(− 2, 0)
10 cm thicker snow compared to long-term mean	Individual (thickening snow cover only)	(0, 10)
2 °C cooler and 10 cm thicker snow compared to long-term means	Compound (cooling)	(− 2, 10)
2 °C warmer compared to the long-term mean	Individual (warming only)	(2, 0)
10 cm thinner snow compared to long-term mean	Individual (thinning snow cover only)	(0, − 10)
2 °C cooler and 10 cm thicker snow compared to long-term means	Compound (warming)	(2,-10)

For cooling, we compare the response to (− 2, 10) at each grid with the summation of the expected FT characteristics under corresponding (− 2, 0) and (0, 10). Similarly for warming, we compare the (2, − 10) with the summation response to (2, 0) and (0, − 10).

**Figure 5:**
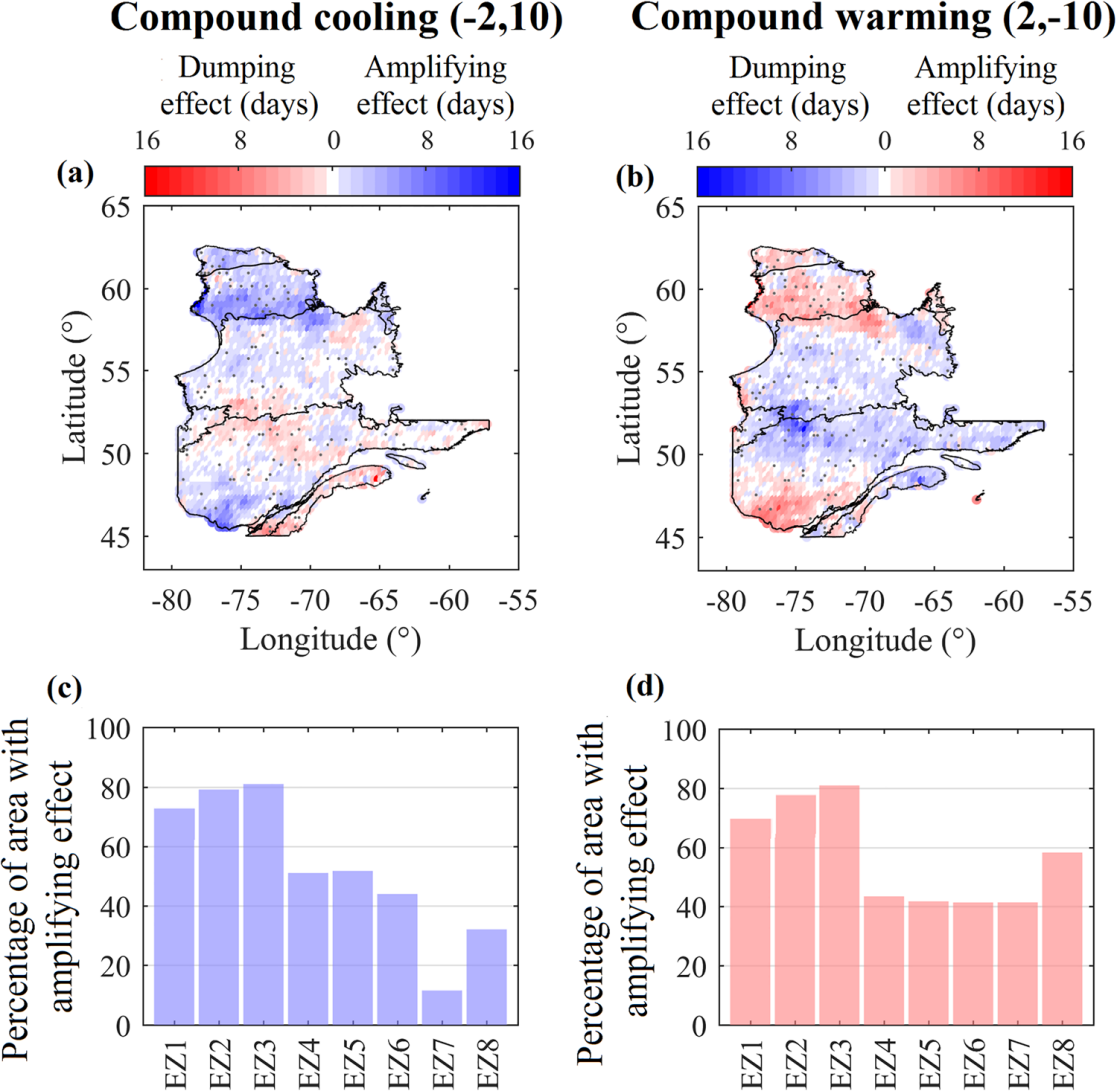
The nonlinear response of *FD*_*year*_ to considered compound cooling (left column) and warming (right column). Nonlinear responses are manifested by amplifying and dumping effects in the length of the frozen period (top row). In each map, black dots show the grids that are excluded from the study due to the existence of autocorrelation in temperature data. In the bottom row, the percentage of areas in each ecozone with an amplified response is shown. This figure is created by the authors using the MATAB R2016a (mathworks.com).

Theoretically at each grid, the variation in *FD*_*year*_ under individual and compound scenarios compared to historical conditions, i.e. $\Delta FD_{year}$, can have one of the following three conditions: (1) linear response, meaning either $\Delta FD_{year} \left( { - 2,10} \right) = \Delta FD_{year} \left( { - 2,0} \right) + \Delta FD_{year} \left( {0,10} \right)$ under cooling and/or $\Delta FD_{year} \left( {2, - 10} \right) = \Delta FD_{year} \left( {2,0} \right) + \Delta FD_{year} \left( {0, - 10} \right)$ under warming; (2) nonlinear dumping response, meaning either $\Delta FD_{year} \left( { - 2,10} \right) < \Delta FD_{year} \left( { - 2,0} \right) + \Delta FD_{year} \left( {0,10} \right)$ under cooling or $\Delta FD_{year} \left( {2, - 10} \right) > \Delta FD_{year} \left( {2,0} \right) + \Delta FD_{year} \left( {0, - 10} \right)$ under warming; or (3) nonlinear amplifying response, meaning either $\Delta FD_{year} \left( { - 2,10} \right) > \Delta FD_{year} \left( { - 2,0} \right) + \Delta FD_{year} \left( {0,10} \right)$ under cooling or $\Delta FD_{year} \left( {2, - 10} \right) < \Delta FD_{year} \left( {2,0} \right) + \Delta FD_{year} \left( {0, - 10} \right)$ under warming. Figure 5 summarizes the results of this analysis. The maps in the top row demonstrate the results related to deviation from the superposition principle at the grid scale under compound cooling (left column) and warming (right column). Under compound cooling, shades of blue and red show amplifying and dumping effects. For compound warming, blue and red colors represent dumping and amplifying effects, respectively. The white color highlights grids in which a linear response is observed. Black dots show the grids where the autocorrelation is present in temperature data and accordingly these grids are excluded from our assessment. The bar charts in the bottom row show the percentage of grids at each ecozone, where amplification in compound response is observed.

Considering this figure, although the amplified response of *FD*_*year*_ to compound changes is observed across the province, it is mainly concentrated in the northern ecozones for both cooling and warming scenarios. Looking at both compound cooling and warming, more than 70% of the area in the three northern ecozones, i.e. Northern Arctic (EZ1), Southern Arctic (EZ2), Arctic Cordillera (EZ3) demonstrate an amplified response. While the expected values for amplifying effect, i.e. $\Delta FD_{year} \left( { - 2,10} \right) - \Delta FD_{year} \left( { - 2,0} \right) - \Delta FD_{year} \left( {0,10} \right)$ in EZ1, EZ2, and EZ3 are 5, 8, and 7 days, respectively, the amplified cooling effect can reach up to 16 days in some grids in northern ecozones. In contrast, the percentage of grids with amplified effect reduces to less than 10% in the EZ7—see Fig. 5c. Similar findings are obtained with regard to the compound warming scenario, although the amplification can also dominate the response of *FD*_*year*_ in the south, e.g. in EZ8 (see Fig. 5d). Having said that, still more amplifications are observed in the three northern ecozones—see Fig. 5d. Although the expected values for amplified warming, i.e. $\Delta FD_{year} \left( {2, - 10} \right) - \Delta FD_{year} \left( {2,0} \right) - \Delta FD_{year} \left( {0, - 10} \right)$ are − 4, − 6, and − 6 days in EZ1, EZ2, and EZ3, the amplified response can get to − 11 days in a grid located in Southern Arctic. This finding has some important implications for the thawing landscape in the northern regions and a wide suite of environmental changes that can be initiated by the amplified response of FT to compound warming. On the one hand, an amplified response can facilitate the access to untapped natural resources of the north and may unleash an opportunity for northern agriculture. On the other hand, however, it intensifies permafrost degradation, which results in emissions of greenhouse gasses^19,20,45^. In contrast, the dumping effect dominates the *FD*_*year*_ response in the southern ecozones, with the exception of EZ8 under compound warming. For both cooling and warming scenarios, the most severe dumping effect is observed in the Atlantic Maritime (EZ7), with the expected and extreme dumping of − 5 and − 9 days under compound cooling as well as 6 and 10 days under compound warming.

**Figure 6:**
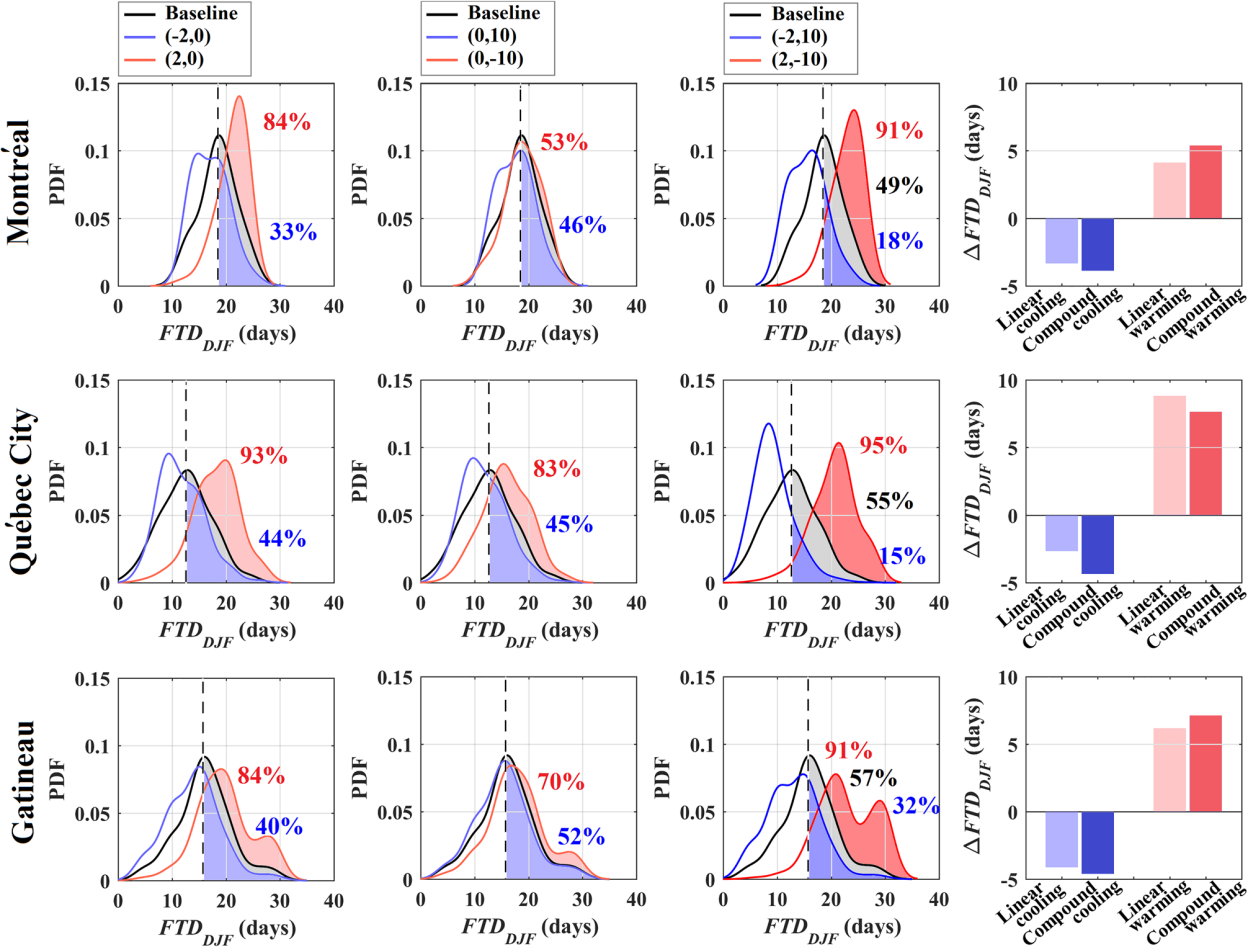
The Probability Density Functions of *FTD*_*DJF*_ along with the likelihood of exceeding the corresponding long-term historical values in Montréal (first row), Québec City (second row), and Gatineau (third row) given opposite individual and compound scenarios for climatic changes, shown in the first, second and third columns from the left, respectively. The nonlinear response of *FTD*_*DJF*_ to considered compound cooling and warming are demonstrated in the far-right column.

The moderate amplification in *FD*_*year*_ response in southern regions can be justified by the lower sensitivity of *FD*_*year*_ to compound changes in temperature and snow depth shown in Fig. 3. Accordingly, we look at the gridded response of *FTD*_*DJF*_ to individual and compound scenarios of change in the three most populated zones of Québec, i.e. Montréal (the grid including 45.5017° N, 73.5673° W), Québec City (the grid including 46.8139° N, 71.2080° W) and Gatineau (the grid including 45.4765° N, 75.7013° W). These three cities are located in the Mixed Wood Plains (EZ8), where the most significant sensitivity in the response of *FTD*_*DJF*_ to compound changes in temperature and snow depth conditions is observed—See Fig. 3. Figure 6 presents the shifts in the mean and exceedance probabilities of *FTD*_*DJF*_, i.e. $\Delta FTD_{DJF}$, given 7 different compound conditions in Montréal (first row), Québec City (second row), and Gatineau (third row). From left to right, the first three columns show the changes in Probability Density Functions (PDFs) of *FTD*_*DJF*_ under (+  2, 0) and (− 2, 0), (0, − 10) and (0, + 10), as well as (+ 2, − 10) and (− 2, + 10), respectively. The right column compares the hypothetical linear response, i.e. $\Delta FTD_{DJF} \left( { - 2,0} \right) + \Delta FTD_{DJF} \left( {0,10} \right)$ in the case of compound cooling and $\Delta FTD_{DJF} \left( {2,0} \right) + \Delta FTD_{DJF} \left( {0, - 10} \right)$ in the case of compound warming, with expected values of $\Delta FTD_{DJF} \left( { - 2,10} \right)$ and $\Delta FTD_{DJF} \left( {2, - 10} \right)$, respectively. The PDFs of *FTD*_*DJF*_ for warming, cooling, and no-change conditions are shown in red, blue, and black colors, respectively; and the expected historical values of *FTD*_*DJF*_ are identified with vertical dashed lines. The highlighted regions in each PDF show likelihoods for *FTD*_*DJF*_ exceeding the long-term historical mean of *FTD*_*DJF*_ under the considered warming (red) or cooling (blue) conditions. In each panel of the right column, light colored bars show hypothetical linear responses under cooling (blue) and warming (red) conditions. Dark colored bars, in contrast, show the expected responses under compound cooling (blue) and warming (red) conditions obtained by the C-vine copula models.

This figure reveals the asymmetric and nonlinear responses of *FTD*_*DJF*_ to individual and compound cooling and warming scenarios in the three most populated cities of Québec. Under individual scenarios, the shift in the mean of *FTD*_*DJF*_ is at least 4 days higher under (2, 0) compared to (− 2, 0), although these shifts are relatively symmetric under (0, − 10) and (0,10). Lower sensitivity to snow depth condition in EZ8 was also shown in Fig. 4(f). Under compound changes, the shift in the mean of *FTD*_*DJF*_ is approximately twice higher under (2, − 10) compared to (− 2, 10). The asymmetric response of *FTD*_*DJF*_ to compound events is also manifested in the exceedance probabilities. For instance, in Montréal the exceedance probability of *FTD*_*DJF*_ changes by 42% under (2, − 10), while the corresponding shift in the exceedance probability under (− 2, 10) is only − 31%. This is another line of evidence for complex responses of FT characteristics to compound cooling and warming. Comparing the expected compound responses with hypothetical linear responses under cooling and warming scenarios, an amplified response to compound cooling is observed in all three cities with expected values of − 2, − 3, and − 1 days in Montréal, Québec City, and Gatineau, respectively. Considering the compound warming scenario, the amplifying effect in Montréal and Gatineau are 2 and 1 days, respectively. It should be noted however that a dumping effect of − 1 day is observed in Québec City under the considered compound warming.

It should be noted that the nonlinearity in the response transcends to exceedance probabilities as well. In Montréal, for instance, the amplifying effects in exceedance probabilities under compound cooling and warming conditions are − 12% and + 3%, respectively. The amplifying response of *FTD*_*DJF*_ to compound warming in a place like Montréal has an important relevance to the regional climate change, manifested by warmer temperatures and less snowfall^56^. Montréal marks one of the most populated regions in Canada with a high concentration of aging infrastructures, vulnerable to increasing *FTD*_*DJF*_. In Montréal, for instance, it is shown that under the historical condition, 216 km length of the city’s watermains need to be replaced and another 2400 km are required to be repaired during the next 20 years^57^. This rate will significantly change under increasing *FTD*_*DJF*_. Current Findings in other cold regions indicate that more swings in FT cycles can translate to multiple million dollars for infrastructure maintenance and repair^47^. In addition, Marshall test of asphalt concrete shows reduced stability by up to 57% when the materials are exposed to only 6 more transient days^58^. This poses an addition vulnerability to aging infrastructures and the built environment^59^.

## Summary and concluding remarks

Landscape FT is arguably the most important land-surface feature in cold regions, controlling physical, biological, and socio-economic processes along with their interactions with other elements of the environment. Historical characteristics of FT, however, are under significant alterations due to climate change, which has important implications for land and resource management in the north. Despite existing understandings of the impact of changing climate on FT cycles, current assessment frameworks are rather limited. On the one hand, in-situ data networks, often used as the main data support for understanding FT responses to changing climate conditions, are rather sparse and therefore fail to provide a synoptic view on different modes of landscape responses. On the other hand, the current projection paradigm based on the use of physically-based land-surface models is incomplete due to several assumptions and/or simplifications in the conceptualization, representation, and parametrization of the interacting processes that determine the state of FT at a given time and space. Our points of innovation are in the use of satellite remote-sensing data in conjunction with a formal statistical technique to overcome some of the above-mentioned limitations, at least at coarser spatial and temporal scales. First, we propose pairing gridded FT characteristics obtained from satellite retrievals with corresponding gridded data of temperature and snow depth, the two most influential hydroclimatic drivers of FT, to study the linkage between FT and climate characteristics. Second, we suggest C-vine copulas to develop a conditional model, with which the impacts of individual and compound changes in hydroclimatic drivers on FT characteristics can be quantified probabilistically.

We showcase the application of this framework in the province of Québec, Canada, a sub-country jurisdiction with an area comparable to Mongolia, the 18th largest country in the world. We show that the simulations obtained by parametrized C-vine copulas can capture the empirical moments of observed FT characteristics as well as the interdependencies between FT, temperature, and snow depth characteristics from 1998 to 2016. The application of the proposed framework for assessing the impact of individual and compound changes in temperature and snow depth reveals some important features of the FT response to climatic changes that have remained rather obscured. First, we highlight an ecozonal differences in the interdependencies between FT characteristics, temperature, and snow depth, pointing at the role of ecosystem conditions and/or latitudinal gradients in regulating the impact of changing climate conditions on FT characteristics. Moreover, a strong north–south divide in the FT response to changing climate conditions is observed. While the impacts of changing climate conditions are manifested in the annual extent of the frozen period in northern regions, they are revealed through alteration in the extent of the transient period during a typical winter season in the south. Through sampling the response surfaces related to *FD*_*year*_ in Northern Arctic and *FTD*_*DJF*_ in Mixed Wood Plains, we also demonstrate a different nature of the response to changing temperature and snow depth conditions between the north and the south. Our results show an asymmetric response to changing temperature and snow depth conditions despite differences in FT variables, regions, and/or spatial scales. In general, where there is a considerable sensitivity in FT response, the alteration due to warming temperature and/or thinning snow depth is more intense compared to corresponding scenarios with cooling temperature and/or thickening snow depth. Closer examination of the FT responses at the grid scale also revealed nonlinear, mainly amplifying, responses of FT characteristics to compound changes in climate conditions. This means that the response of FT to compound changes in temperature and snow depth is often more severe than the cumulative response of FT to changes in temperature and snow depth individually. These amplifying impacts can result in up to two weeks of alterations in the frozen period during a typical year in the north, and up to one week change in the transient period during a typical winter season in the three most populated regions of Québec, i.e., Montréal, Québec City, and Gatineau. While the amplified shortening in the annual frozen period in the north can result in unprecedented changes in the northern environment under compound warming, the similar climatic condition can result in additional stress to already aging infrastructures in the south. We show that the amplified response is not only observed in the expected values of the transient period in a typical winter, but also in the likelihood of exceeding the long-term historical values.

Our proposed methodology is generic and can be applied globally. We encourage inspecting the asymmetry, nonlinearity, and spatial variability of FT response to changing climate conditions in other Canadian regions and globally. In the context of Québec, landscape responses to freeze and thaw can have a wide range of implications from endangering already aging and vulnerable community infrastructures to substantial environmental changes due to amplified rates of permafrost degradation. The latter can be even globally relevant due to massive land-induced carbon emissions. Facing these challenges requires integrated and inclusive approaches that are supported by relevant and evidence-based scientific information. In that line, we will soon report the application of the proposed framework for estimating future FT characteristics in Québec using available downscaled climate projections: *donc à bientôt!*

## Methods

### Data support

We use the global landscape FT Earth System Data Record (FT-ESDR) from the National Snow and Ice Data Center (https://doi.org/10.5067/MEASURES/CRYOSPHERE/nsidc-0477.004). This dataset includes the daily state of soil at the spatial resolution of 25 × 25 km^2^ over the period of 1979–2017. The remotely-sensed brightness temperature along with an improved retrieval algorithm are used to classify FT states into four distinct classes of frozen (AM and PM frozen), non-frozen (AM and PM thawed), transitional (AM frozen and PM thawed), and inverse-transitional (AM thawed and PM frozen)^60^. We categorize transitional and inverse-transitional states into one combined transient state that shows whether landscape switches between the frozen and thawed conditions in a diurnal cycle or not. Knowing the gridded daily states of FT, two critical FT characteristics, namely *FD*_*year*_ and *FTD*_*DJF*_, can be extracted at the grid scale or each ecozone, and accordingly paired with corresponding gridded temperature and snow depth data. For air temperature data, we use the Global Meteorological Forcing Dataset (GMFD) provided by Princeton University available at https://hydrology.princeton.edu/data.pgf.php. GMFD dataset is constructed by blending the reanalysis data from the National Centers for Environmental Prediction, National Center for Atmospheric Research with a group of recent global observation-based data^37^. GMFD provides daily maximum and minimum air temperature at 0.25° × 0.25° for the period of 1948–2016. The daily mean temperature is calculated by averaging the daily maximum and minimum temperature at each grid. Monthly snow depth data are obtained from the Canadian Meteorological Center (CMC; https://doi.org/10.5067/W9FOYWH0EQZ3). CMC dataset is constructed by combining the information from in-situ snow depth measurements with optimal interpolation results of a simple physical snow accumulation and melt model^38^. The data is provided for the period of 1998–2020 at 24 × 24 km^2^ across the northern hemisphere.

As the grid size and centroid locations of the three considered data sources are not the same, we implement *k*-nearest neighbor interpolation^61^ to re-grid the three data sets into a unique spatial scale and over the common period of 1998–2016. In brief, the *k*-nearest neighbor is a non-parametric approach to estimate a variable in a given point in time and space based on its neighboring values. The nearest neighbors are identified as those with the smallest Euclidian distance to the center of a reference grid, here the grid of FT, to which climate data are re-gridded. After finding the optimal nearest neighbors, a weighted averaging is applied to rescale the variables. The weight function gains its maximum value where the distance from the interpolated point is zero and decreases as the distance increases^62^. By implementing some numerical experiments, we find *k* = 4 as the optimal number of nearest neighbors to achieve the highest accuracy in modeling the mean and standard deviation of temperature and snow depth over different ecozones of Québec. The re-gridded monthly mean temperature and monthly mean snow depth are then matched with the corresponding *FD*_*year*_ and *FTD*_*DJF*_ at the same temporal and spatial scales. Before developing dependence models, we investigate the existence of autocorrelation in temperature, snow depth, and FT characteristics and exclude those grids in which the autocorrelation is significant. We find that this is the case in less than 17% of grids, and only for annual temperature. These grids are excluded from our analysis.

### Proposed copula-based impact assessment framework

To model the joint dependencies between FT characteristics, air temperature, and snow depth, trivariate copulas can be used. Based on the Sklar’s Theorem, the joint dependencies between FT characteristics ($FT$), mean temperature ($\overline{T }$), and snow depth ($SD$) can be described as:

1 $$ F\left( {FT, \overline{T},SD} \right) = C\left( {F_{1} \left( {FT} \right),F_{2} \left( { \overline{T}} \right),F_{3} \left( {SD} \right)} \right) $$

where ${F}_{1}(FT)$, ${F}_{2}(\overline{T })$ and ${F}_{3}(SD)$ are the Cumulative Distribution Functions (CDFs) for $FT$, $\overline{T }$ and $SD$, respectively and $C$ is the trivariate copula function^63^. Among different alternative multivariate copulas, we employ canonical vine (C-vine) to represent joint distribution between the three above-mentioned variables in Eq. (1)^64^. In brief, C-vine copulas decompose a high-dimensional joint distribution into a $d(d-1)/2$ bivariate pairs of copulas arranged into $(d-1)$ trees; and accordingly, the joint distribution between $FT$, $\overline{T }$ and $SD$ can be described as:

2 $$ f\left( {FT,\overline{T},SD} \right) = f_{2} \left( {\overline{T}} \right)f_{3|2} (SD|\overline{T})f_{1|2,3} (FT|\overline{T},SD) $$

where $f\left(.\right)$ is the marginal PDFs and the conditional distributions that can be estimated as^65^:

3 $$ f_{3|2} (SD|\overline{T}) = \frac{{f\left( {SD,\overline{T}} \right)}}{{f\left( {\overline{T}} \right)}} = \frac{{c_{2,3} \left( {F_{2} \left( {\overline{T}} \right),F_{3} \left( {SD} \right)} \right)f_{2} \left( {\overline{T}} \right)f_{3} \left( {SD} \right)}}{{f_{2} \left( {\overline{T}} \right)}} = c_{2,3} \left( {F_{2} \left( {\overline{T}} \right),F_{3} \left( {SD} \right)} \right)f_{3} \left( {SD} \right) $$

and

4 $$ \begin{aligned} & f_{1|2,3} \left( {FT{|}\overline{T},SD} \right) = \frac{{f\left( {FT,SD|\overline{T}} \right)}}{{f\left( {SD|\overline{T}} \right)}} = \frac{{c_{1,3|2} \left( {F\left( {FT|\overline{T}} \right),F\left( {SD|\overline{T}} \right)} \right)f\left( {FT|\overline{T}} \right)f\left( {SD|\overline{T}} \right)}}{{f\left( {SD|\overline{T}} \right)}} \\ & \quad = c_{1,3|2} \left( {F\left( {FT|\overline{T}} \right),F\left( {SD|\overline{T}} \right)} \right)c_{1,2} \left( {F_{1} \left( {FT} \right),F_{2} \left( {\overline{T}} \right)} \right)f_{1} \left( {FT} \right) \\ \end{aligned} $$

where *c*(·) is the 3-dimensional copula density. As a result, the three dimensional joint density can be represented in terms of bivariate copulas as the following^66^:

5 $$ f\left( {FT,\overline{T},SD} \right) = f_{1} \left( {FT} \right).f_{2} \left( {\overline{T}} \right).f_{3} \left( {SD} \right).c_{2,1} .c_{2,3} .c_{1,3|2} $$

where ${c}_{\mathrm{2,1}}({F}_{2}\left(\overline{T }\right),{F}_{1}\left(FT\right))$ and ${c}_{\mathrm{2,3}}({F}_{2}\left(\overline{T }\right),{F}_{3}\left(SD\right))$ are simply written as ${c}_{\mathrm{2,1}}$ and ${c}_{\mathrm{2,3}}$; and the conditional pairwise copulas between ${F}_{1}\left(FT\right)$ and ${F}_{3}\left(SD\right)$ conditional to ${F}_{2}\left(\overline{T }\right)$, i.e. ${c}_{\mathrm{1,3}|2}\left({F}_{1}\left(FT\right),{F}_{3}\left(SD\right)|{F}_{2}\left(\overline{T }\right)\right)$ is shown by ${c}_{\mathrm{1,3}|2}$. In addition, ${c}_{\mathrm{2,1}}$, ${c}_{\mathrm{2,3}}$ and ${c}_{\mathrm{1,3}|2}$ are the densities of bivariate pairs. Having the C-vine copulas, the probability distribution of FT characteristics due to different quantitative changes in temperature and snow depth can be obtained through conditional modeling as:

6 $$ h = F\left( {FT{|}\overline{T},SD} \right) = \frac{{\partial C_{1,3|2} \left( {F\left( {FT|\overline{T}} \right),F\left( {SD|\overline{T}} \right)} \right)}}{{\partial F\left( {SD|\overline{T}} \right)}} $$

where $F\left(FT|\overline{T },SD\right)$ is the conditional distribution function. Moreover,

7 $$ F\left( {FT{|}\overline{T}} \right) = h\left( {FT|\overline{T}} \right) = \frac{{\partial C_{1,2} \left( {F\left( {FT} \right),F\left( {\overline{T}} \right)} \right)}}{{\partial F\left( {\overline{T}} \right)}} $$

and

8 $$ F\left( {SD{|}\overline{T}} \right) = h\left( {SD|\overline{T}} \right) = \frac{{\partial C_{3,2} \left( {F\left( {SD} \right),F\left( {\overline{T}} \right)} \right)}}{{\partial F\left( {\overline{T}} \right)}} $$

Using Eqs. (7) and (8), Eq. (6) can be rewritten as:

9 $$ h = F\left( {FT{|}\overline{T},SD} \right) = h\left[ {h\left( {FT|\overline{T}} \right)|h\left( {SD|\overline{T}} \right)} \right] $$

The estimated CDF of characteristics can be back transformed to the original quantile space using the inverse CDF function, assuming empirical distributions for $FT$, $\overline{T }$ and $SD$ at each ecozone. The inverse form of *h*-function given in Eq. (9) is applied for this purpose. To extract the probability distribution of FT characteristics, a Monte Carlo-based simulation is adopted by generating 1000 random set of FT characteristics under known values of temperature and snow depth^67^. Given random uniform numbers of $\varepsilon$, given FT characteristics can be sampled as:

10 $$ FT = F^{ - 1} \left\{ {h^{ - 1} \left[ {\left( {h^{ - 1} \left( {\varepsilon |h\left( {SD{|}\overline{T}} \right)} \right)} \right)|\overline{T}} \right]} \right\} $$

Computer models for conducting these simulations are developed in *CRAN R* with the use of packages of *VineCopula*, *CDVine*, and *copula*^68–70^. Tree structures for C-vine copulas are selected based on the maximum spanning tree algorithm, in which copula parameters are chosen with respect to the interdependencies between pairwise variables^71^. A set of well-known parametric copula families (i.e. Frank, Gaussian, Student t, Clayton, Gumbel, and Joe) are used to develop, falsify and select pairwise bivariate copulas. The formulations of these copulas are provided in detail in other sources^72^. These copulas are parameterized using the Maximum log-Likelihood Method and considering empirical margins along with the Bayesian information criteria as the Goodness-of-Fit measure^73^. The pool of developed structures at each ecozone are then compared and evaluated based on their capability in representing the marginal FT characteristics and preserving empirical dependencies between a given FT characteristic and $\overline{T }$ or *SD*. Dependencies are quantified using the Kendall’s tau non-parametric dependence measure and the associated hypothetical test^74^. The best C-vine copula structure is then used for conditioning the control of compounding changes in temperature and snow depth on FT characteristics. The one-way ANalysis Of VAriance (ANOVA) with Bonferroni correction is used to formally examine any change in the estimated dependencies across different spatial regions^75^.

## Data Availability

All data used in this study along with additional data related to elevation, land-use, and land-cover, as well as existing in-situ climatic and hydrometric networks are available through the Cold Region Data Accessibility Portal for Québec (CRDAP – QC; http://wscc.encs.concordia.ca/home.html).

## References

[1] Frauenfeld OW, Zhang T, Barry RG (2004). Interdecadal changes in seasonal freeze and thaw depths in Russia. J. Geophys. Res..

[2] Mccauley CA, White DM, Lilly MR, Nyman DM (2002). A comparison of hydraulic conductivities, permeabilities and infiltration rates in frozen and unfrozen soils. Cold Reg. Sci. Technol..

[3] Jones BM (2011). Modern thermokarst lake dynamics in the continuous permafrost zone, northern Seward Peninsula, Alaska. J. Geophys. Res. Biogeosci..

[4] Jansson JK, Taş N (2014). The microbial ecology of permafrost. Nat. Rev. Microbiol..

[5] Margesin R, Neuner G, Storey KB (2007). Cold-loving microbes, plants, and animals—Fundamental and applied aspects. Naturwissenschaften.

[6] Hjort J (2018). Degrading permafrost puts Arctic infrastructure at risk by mid-century. Nat. Commun..

[7] Plaza C (2019). Direct observation of permafrost degradation and rapid soil carbon loss in tundra. Nat. Geosci..

[8] Henry HAL (2008). Climate change and soil freezing dynamics: Historical trends and projected changes. Clim. Change.

[9] Wu Q, Zhang T (2010). Changes in active layer thickness over the Qinghai-Tibetan Plateau from 1995 to 2007. J. Geophys. Res. Atmos..

[10] Schuur EAG (2009). The effect of permafrost thaw on old carbon release and net carbon exchange from tundra. Nature.

[11] Iwata Y, Hayashi M, Suzuki S, Hirota T (2010). Effects of snow cover on soil freezing, water movement, and snowmelt infiltration: A paired plot experiment. Water Resour. Res..

[12] Chen L (2020). Influences of forest cover on soil freeze-thaw dynamics and greenhouse gas emissions through the regulation of snow regimes: A comparison study of the farmland and forest plantation. Sci. Total Environ..

[13] Zhang, T. Influence of the seasonal snow cover on the ground thermal regime: An overview. *Rev. Geophys.* **43**, (2005).

[14] Liu J, Wang S, Yu S, Yang D, Zhang L (2009). Climate warming and growth of high-elevation inland lakes on the Tibetan Plateau. Glob. Planet. Change.

[15] Williams CM, Henry HAL, Sinclair BJ (2015). Cold truths: How winter drives responses of terrestrial organisms to climate change. Biol. Rev..

[16] Park H, Kim Y, Kimball JS (2016). Widespread permafrost vulnerability and soil active layer increases over the high northern latitudes inferred from satellite remote sensing and process model assessments. Remote Sens. Environ..

[17] Meshesha TW, Wang J, Melaku ND (2020). Modelling spatiotemporal patterns of water quality and its impacts on aquatic ecosystem in the cold climate region of Alberta, Canada. J. Hydrol..

[18] Gibson CM, Brinkman T, Cold H, Brown D, Turetsky M (2021). Identifying increasing risks of hazards for northern land-users caused by permafrost thaw: Integrating scientific and community-based research approaches. Environ. Res. Lett..

[19] Wagner-Riddle C (2017). Globally important nitrous oxide emissions from croplands induced by freeze-thaw cycles. Nat. Geosci..

[20] Schaefer K, Lantuit H, Romanovsky VE, Schuur EAG, Witt R (2014). The impact of the permafrost carbon feedback on global climate. Environ. Res. Lett..

[21] Andrews TD (2016). Permafrost thaw and Aboriginal cultural landscapes in the Gwich’in Region, Canada. APT Bull. J. Preserv. Technol..

[22] *Atlas of Canada*. (Natural Resources Canada, 2016).

[23] Hatami, S. & Nazemi, A. Temperature Controls of the Freeze and Thaw Patterns in Québec.” In Canadian Society for Civil Engineering (pp. 1–7). Laval. https://www.csce.ca/elf/apps/CONFERENCEVIEWER/conferences/2019/pdfs/PaperPDFversion_7_0513041747.pdf (2019) (retrieved on 2021–11–22).

[24] Hatami, S., & Nazemi, A. The Compound Impacts of Changing Temperature and Snow Cover on Freeze and Thaw Patterns across Québec. In Geo-Extreme 2021 (pp. 368–376), 10.1061/9780784483701.035.

[25] Amir Jabbari A, Nazemi A (2019). Alterations in Canadian hydropower production potential due to continuation of historical trends in climate variables. Resources.

[26] Fang X, Luo S, Lyu S (2019). Observed soil temperature trends associated with climate change in the Tibetan Plateau, 1960–2014. Theor. Appl. Climatol..

[27] Guo W (2018). Agricultural and forest meteorology vegetation can strongly regulate permafrost degradation at its southern edge through changing surface freeze-thaw processes. Agric. For. Meteorol..

[28] Zhang X, Wu Y, Zhai E, Ye P (2021). Coupling analysis of the heat-water dynamics and frozen depth in a seasonally frozen zone. J. Hydrol..

[29] Walvoord MA, Kurylyk BL (2016). Hydrologic impacts of thawing permafrost—A review. Vadose Zo. J..

[30] Zhang K (2007). Sensitivity of pan-Arctic terrestrial net primary productivity simulations to daily surface meteorology from NCEP-NCAR and ERA-40 reanalyses. J. Geophys. Res..

[31] Tucker CJ (2005). An extended AVHRR 8-km NDVI dataset compatible with MODIS and SPOT vegetation NDVI data. Int. J. Remote Sens..

[32] Favre A, Adlouni SE, Perreault L, Thie N, Bobe B (2004). Multivariate hydrological frequency analysis using copulas. Water Resour. Res..

[33] Nelsen RB (2007). An Introduction to Copulas.

[34] Zaerpour M, Papalexiou SM, Nazemi A (2021). Informing stochastic streamflow generation by large-scale climate indices at single and multiple sites. Adv. Water Resour..

[35] Hatami S, Nazemi A (2021). A statistical framework for assessing temperature controls on landscape freeze–thaw: Application and implications in Québec, Canada (1979–2016). J. Hydrol..

[36] Kim Y, Kimball JS, McDonald KC, Glassy J (2011). Developing a global data record of daily landscape freeze/thaw status using satellite passive microwave remote sensing. Geosci. Remote Sens. IEEE Trans..

[37] Sheffield J, Goteti G, Wood EF (2006). Development of a 50-year high-resolution global dataset of meteorological forcings for land surface modeling. J. Clim..

[38] Brown, R. D. & Brasnet, B. *Canadian Meteorological Centre (CMC) Daily Snow Depth Analysis Data, Version 1*. *Boulder, Colorado USA. NASA National Snow and Ice Data Center Distributed Active Archive Center* (2010). 10.5067/W9FOYWH0EQZ3

[39] Schultz J (2005). The ecozones of the world.

[40] Wiken, E. B. *Terrestrial Ecozones of Canada* (1986).

[41] Sorensen PO (2018). Winter soil freeze-thaw cycles lead to reductions in soil microbial biomass and activity not compensated for by soil warming. Soil Biol. Biochem..

[42] Zhang P, Wittmann FH, Vogel M, Müller HS, Zhao T (2017). Influence of freeze-thaw cycles on capillary absorption and chloride penetration into concrete. Cem. Concr. Res..

[43] Sharma S, Szele Z, Schilling R, Munch JC, Schloter M (2006). Influence of freeze-thaw stress on the structure and function of microbial communities and denitrifying populations in soil. Appl. Environ. Microbiol..

[44] Poppel, B., Fægteborg, M., Siegstad, O. & Snyder, H. T. The Arctic as a ‘hotspot’ for natural extraction and global warming. *Econ. North* 129–135 (2015).

[45] Schuur E (2015). Climate change and the permafrost carbon feedback. Nature.

[46] Kimiaghalam N, Goharrokhi M, Clark SP, Ahmari H (2015). A comprehensive fluvial geomorphology study of riverbank erosion on the Red River in Winnipeg, Manitoba, Canada. J. Hydrol..

[47] Melvin AM (2017). Climate change damages to Alaska public infrastructure and the economics of proactive adaptation. Proc. Natl. Acad. Sci..

[48] Nazemi A, Wheater HS, Chun KP, Elshorbagy A (2013). A stochastic reconstruction framework for analysis of water resource system vulnerability to climate-induced changes in river flow regime. Water Resour. Res..

[49] Liu Z (2018). A framework for exploring joint effects of conditional factors on compound floods. Water Resour. Res..

[50] Carpino OA, Berg AA, Quinton WL, Adams JR (2018). Climate change and permafrost thaw-induced boreal forest loss in northwestern Canada. Environ. Res. Lett..

[51] Lewkowicz AG, Way RG (2019). Extremes of summer climate trigger thousands environment. Nat. Commun..

[52] Roseen RM, Ballestero TP, Houle JJ, Briggs JF, Houle KM (2012). Water quality and hydrologic performance of a porous asphalt pavement as a storm-water treatment strategy in a cold climate. J. Environ. Eng..

[53] Doughty M, Eyles N, Eyles C (2013). High-resolution seismic reflection profiling of neotectonic faults in Lake Timiskaming, Timiskaming Graben, Ontario-Quebec, Canada. Sedimentology.

[54] Mazdiyasni O, AghaKouchak A (2015). Substantial increase in concurrent droughts and heatwaves in the United States. Proc. Natl. Acad. Sci. U. S. A..

[55] Chiang F, Mazdiyasni O, AghaKouchak A (2018). Amplified warming of droughts in southern United States in observations and model simulations. Sci. Adv..

[56] Hatami, S., Nazemi, A. & Amirjabbari, A. Evolving Trends of Rain over Precipitation in Canadian Cold Season During the late 20th Century. in Canadian Society for Civil Engineering (pp. 1–5), Laval. https://www.csce.ca/elf/apps/CONFERENCEVIEWER/conferences/2019/pdfs/PaperPDFversion_8_0513041903.pdf (2019) (retrieved on 2021–11–22).

[57] Zangenehmadar Z, Moselhi O, Golnaraghi S (2020). Optimized planning of repair works for pipelines in water distribution networks using genetic algorithm. Eng. Rep..

[58] Özgan E, Serin S (2013). Cold regions science and technology investigation of certain engineering characteristics of asphalt concrete exposed to freeze–thaw cycles. Cold Reg. Sci. Technol..

[59] Farran M, Zayed T (2009). Comparative analysis of life-cycle costing for rehabilitating infrastructure systems. J. Perform. Constr. Facil..

[60] Kim Y, Kimball JS, Glassy J, Du J (2017). An extended global Earth system data record on daily landscape freeze–thaw status determined from satellite passive microwave remote sensing. Earth Syst. Sci. Data.

[61] Cover TM, Hart PE (1967). Nearest neighbor pattern classification. IEEE Trans. Inf. theory.

[62] Fekete BM, Vrsmarty CJ, Lammers RB (2001). Scaling gridded river networks for macroscale hydrology: Development, analysis, and control of error. Water Resour. Res..

[63] Sklar M (1959). Fonctions de repartition an dimensions et leurs marges.

[64] Bedford BYTIM, Cooke RM, Vines, (2002). A new graphical model for dependent random variables author. Ann. Stat..

[65] Aas K, Czado C, Frigessi A, Bakken H (2009). Pair-copula constructions of multiple dependence. Insur. Math. Econ..

[66] Joe H (1997). Multivariate Models and Multivariate Dependence Concepts.

[67] Roy T, Gupta H (2021). How certain are our uncertainty bounds? Accounting for sample variability in Monte Carlo-based uncertainty estimates. Environ. Model. Softw..

[68] Schepsmeier, U. *et al.* Package ‘VineCopula’. *R Packag. version* **2**, (2015).

[69] Brechmann EC, Schepsmeier U (2013). CDVine: Modeling dependence with C- and D-vine Copulas in R Eike. J. Stat. Softw..

[70] Yan J (2007). Enjoy the joy of copulas: With a package copula. J. Stat. Softw..

[71] Dißmann J, Brechmann EC, Czado C, Kurowicka D (2013). Selecting and estimating regular vine copulae and application to financial returns. Comput. Stat. Data Anal..

[72] Nelsen RB (2006). An Introduction to Copulas.

[73] Sadegh M, Ragno E, Aghakouchak A (2017). Multivariate Copula analysis toolbox (MvCAT): Describing dependence and underlying uncertainty using a Bayesian framework. Water Resour. Res..

[74] Kendall AMG (1938). A new measure of rank correlation. Oxford Univ. Press Behalf Biometrika Trust.

[75] Nazemi A, Zaerpour M, Hassanzadeh E (2020). Uncertainty in bottom-up vulnerability assessments of water supply systems due to regional streamflow generation under changing conditions. J. Water Resour. Plan. Manag..

